# One-Year-Old Precocious Chinese Mitten Crab Identification Algorithm Based on Task Alignment

**DOI:** 10.3390/ani14142128

**Published:** 2024-07-21

**Authors:** Hao Gu, Dongmei Gan, Ming Chen, Guofu Feng

**Affiliations:** Key Laboratory of Fisheries Information, Ministry of Agriculture and Rural Affairs, Shanghai Ocean University, Hucheng Ring Road 999, Shanghai 201306, China; m220901555@st.shou.edu.cn (D.G.); gffeng@shou.edu.cn (G.F.)

**Keywords:** aquaculture seedling, target recognition, machine learning, deformable convolutional networks, adaptive spatial feature fusion

## Abstract

**Simple Summary:**

Simple Summary: We developed R-TNET, a detection model tailored to identify one-year-old sexually precocious crabs. By addressing issues like subtle classification features and overlapping masks, the model was able to accurately identify and localize juvenile crabs on the validation data, and the mean average precision reached 88.78%. This effect meets practical needs and can be applied in the field of intelligent sorting, thus improving non-destructive technology in aquaculture.

**Abstract:**

The cultivation of the Chinese mitten crab (*Eriocheir sinensis*) is an important component of China’s aquaculture industry and also a field of concern worldwide. It focuses on the selection of high-quality, disease-free juvenile crabs. However, the early maturity rate of more than 18.2% and the mortality rate of more than 60% make it difficult to select suitable juveniles for adult culture. The juveniles exhibit subtle distinguishing features, and the methods for differentiating between sexes vary significantly; without training from professional breeders, it is challenging for laypersons to identify and select the appropriate juveniles. Therefore, we propose a task-aligned detection algorithm for identifying one-year-old precocious Chinese mitten crabs, named R-TNET. Initially, the required images were obtained by capturing key frames, and then they were annotated and preprocessed by professionals to build a training dataset. Subsequently, the ResNeXt network was selected as the backbone feature extraction network, with Convolutional Block Attention Modules (CBAMs) and a Deformable Convolution Network (DCN) embedded in its residual blocks to enhance its capability to extract complex features. Adaptive spatial feature fusion (ASFF) was then integrated into the feature fusion network to preserve the detailed features of small targets such as one-year-old precocious Chinese mitten crab juveniles. Finally, based on the detection head proposed by task-aligned one-stage object detection, the parameters of its anchor alignment metric were adjusted to detect, locate, and classify the crab juveniles. The experimental results showed that this method achieves a mean average precision (mAP) of 88.78% and an F1-score of 97.89%. This exceeded the best-performing mainstream object detection algorithm, YOLOv7, by 4.17% in mAP and 1.77% in the F1-score. Ultimately, in practical application scenarios, the algorithm effectively identified one-year-old precocious Chinese mitten crabs, providing technical support for the automated selection of high-quality crab juveniles in the cultivation process, thereby promoting the rapid development of aquaculture and agricultural intelligence in China.

## 1. Introduction

*Eriocheir sinensis* (Crabidae, Decapoda, Crustacea) is an important aquaculture target in China, and its yearly production amounts to more than 0.7 million tons [1]. It can be seen that it also plays a crucial role in the aquaculture industry worldwide. Since the 1980s, when artificial seawater preparation technology [2] was adopted to break through the breeding problem of *Eriocheir sinensis*, China has established a solid industrial chain from juvenile crab breeding [3], crab breed cultivation, adult crab breeding, and processing to export, which has become an important way to increase income in agriculture and make fishermen rich. However, in the process of crab seed breeding, inbreeding [4] due to artificial reproduction, the use of small-size parents for seedling breeding, introductions between different aquatic systems, and disorderly breeding have caused serious degradation of germplasms and the mixing of river crab breeding populations. This has had a significant impact on breeding performance and has brought greater harm to the river crab aquaculture industry [5].

The issue of precocious maturity in one-year-old Chinese mitten crabs has long hindered development in the industry [6]. Typically, it refers to individuals weighing over 20 g with developed gonads, known as “old man crabs”. In natural habitats like the Yangtze River Basin, around 5% to 10% exhibit precocious maturity, while in controlled environments, rates can soar to 18.2% to 98.0% due to factors like temperature and nutrition [7]. Cultivating these precocious crabs leads to high adult mortality rates (60% to 90%) and diminished market value due to their small size [8]. Consequently, reducing their proportion is vital for productivity and profitability. However, despite various theories, no definitive method exists to eliminate precocious maturity [9]. Hence, rigorous screening during species selection is crucial.

Traditionally, identifying one-year-old precocious Chinese mitten crabs relies on manual observation, using visual cues such as differences in abdomen morphology and velvet characteristics [10]. However, this method is inefficient and prone to errors. With the advancement of computer vision technology, employing image processing and machine learning algorithms has emerged as a promising approach. These techniques enable automatic analysis of large image datasets, accurately identifying subtle biometric differences without human intervention [11]. In summary, computer vision-based detection methods for precocious crabs offer practical feasibility and advantages [12].

Recent advancements in aquatic research have focused on the identification and localization of river crabs, leading to significant developments in technology. The use of the YOLOv3 algorithm on underwater images has provided reliable data for automated baiting boats [13], supporting precision feeding strategies through highly accurate predictions of crab biomass and location. Further refinements include the adoption of the lightweight Efficient Net, enabling the model to operate on feeding devices with minimal storage requirements while maintaining high detection accuracy and completeness [14]. This is particularly useful for resource-constrained environments like automated baiting vessels.

Additionally, the focus has shifted towards the sex classification of crabs to optimize the benefits during the selling stage [15]. Techniques leveraging the ResNet50 network to analyze abdominal features have demonstrated high accuracy and reliability in distinguishing between male and female crabs [16]. However, the effectiveness of these algorithms can be compromised in production environments with complex backgrounds, where high-quality image data are crucial for accurate classification [17]. To address this, Chen et al. [18] proposed a lightweight crab detection and gender classification method using the improved YOLOv4, achieving remarkable accuracy. Gu et al. [19] constructed a dataset for Chinese mitten crab fries and achieved high accuracy in gender classification using an enhanced Faster R-CNN network, even with small samples and inconspicuous gender characteristics.

Identifying one-year-old precocious mitten crabs is challenging due to their juvenile stage, small size, and potential overlap. These crabs exhibit fewer distinct classification features compared to adult gender classification, with distinguishing features not confined to the abdominal region. To ensure accurate detection, deep learning-based target detection algorithms are utilized. These algorithms fall into two categories [20]: region-based methods like the R-CNN series, which generate candidate regions for feature extraction and classification but are computationally intensive, and single-stage detection methods like YOLO and TOOD [21], which directly predict the target category and location, offering more efficiency. Given the challenges of uneven size, feature overlap, and complex backgrounds, the TOOD algorithm is selected as the benchmark model for detecting one-year-old precocious crabs in this study. The main contributions of this paper are as follows:

(1) Introducing ResNeXt as the backbone network, embedding a CBAM in the residual block, and using variable convolution in the last residual block to enhance feature extraction for sexually precocious Chinese mitten crabs.
(2) Combining the ASFF module with the traditional FPN for integrated training to solve feature fusion mismatches and reduce the loss of small-target information.
(3) Conducting experiments on the anchor alignment metric formula to optimize the values of *p* and *q*, improving accuracy by adjusting the proportion of *s*.

## 2. Materials and Methods

### 2.1. Data Acquisition

#### 2.1.1. Crab Dataset

**Figure 1:**
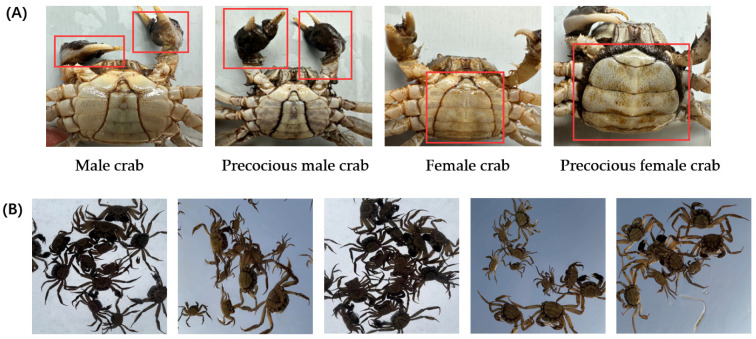
The presentation of crab datasets. (**A**) Difference between precocious crabs and normal samples. (**B**) Raw dataset with unclear features.

Creating datasets is crucial for deep learning-based target detection [22]. Given the limited availability of datasets for identifying one-year-old precocious Chinese mitten crabs, this study independently constructs one using images primarily from artificially cultured crabs in Yangcheng Lake. These images were collected over ten months, reflecting the morphological characteristics of one-year-old precocious crabs (Figure 1). The early male crabs have more velvet in the pincer area, while the female crabs have fuller and rounder abdomens (Figure 1A). To ensure relevance to practical needs, the images were captured during the critical period for crab juvenile selection. We placed one-year-old precocious crabs and normal samples into transparent tanks and photographed them from bottom to top using a camera. While variations in background and backlighting affected abdominal feature visibility, occlusion and overlapping further complicated image clarity. The preprocessing and data enhancement techniques addressed these challenges in subsequent steps.

#### 2.1.2. Data Preprocessing

**Figure 2:**
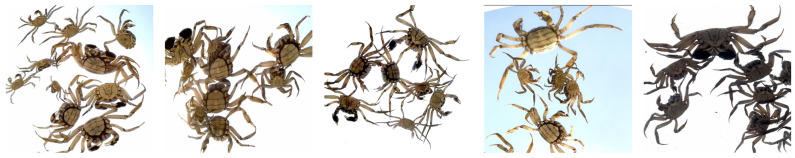
Preprocessed dataset with rich features.

This study acquired a total of 700 high-resolution images (4032 × 3024 pixels in JPG format) of one-year-old precocious Chinese mitten crabs, including 846 males and 928 females. These images were labeled using Labelme 3.16.5 software [23]. To enhance the visibility of features obscured by background effects, the exposure of the original images was increased. Adjusting the exposure to 90% significantly improved the visibility of the abdominal features, facilitating easier distinction of the crabs (Figure 2).

#### 2.1.3. Data Augmentation

In computer vision, deep convolutional neural networks are highly regarded for object detection tasks. However, small sample datasets often lead to poor model generalization and increased risk of overfitting [24]. Our labeled dataset revealed a significant lack of target frames for one-year-old precocious Chinese mitten crabs, impacting the experimental accuracy. To mitigate this, fine-tuning and optimizing the model parameters for different targets were crucial to minimize the global loss function and improve the detection efficiency. Given the challenge of collecting extensive data, enhancing the dataset through brightness adjustment, rotation, and scaling was undertaken. This resulted in a dataset of 3928 images, including 16,820 early crabs, with a ratio of 9:1 between the training and validation sets (Table 1).

### 2.2. Experimental Environment

In order to implement the methods in this paper, all experiments were carried out under the Ubuntu operating system and PyTorch deep learning framework. The server CPU model was Intel(R) Core i9-11900KF, 64 GB RAM, and an NVIDIA 3090 GPU (24 GB). The deep learning environment was composed of PyTorch 1.6.1, Python 3.7, CUDA 11.7, and cuDNN 8.5.0.

### 2.3. Overview of TOOD Framework

The Task-Aligned One-Stage Object Detection (TOOD) framework is an advanced single-stage object detection method designed to improve detection accuracy and speed [25]. It addresses the limitations of traditional methods by enhancing task alignment and optimization efficiency. TOOD’s key innovation is its task-aligned optimization strategy, which introduces a task alignment mechanism throughout feature extraction, candidate frame generation, classification, and localization. This strategy effectively coordinates classification and localization, significantly improving detection performance.

**Figure 3:**
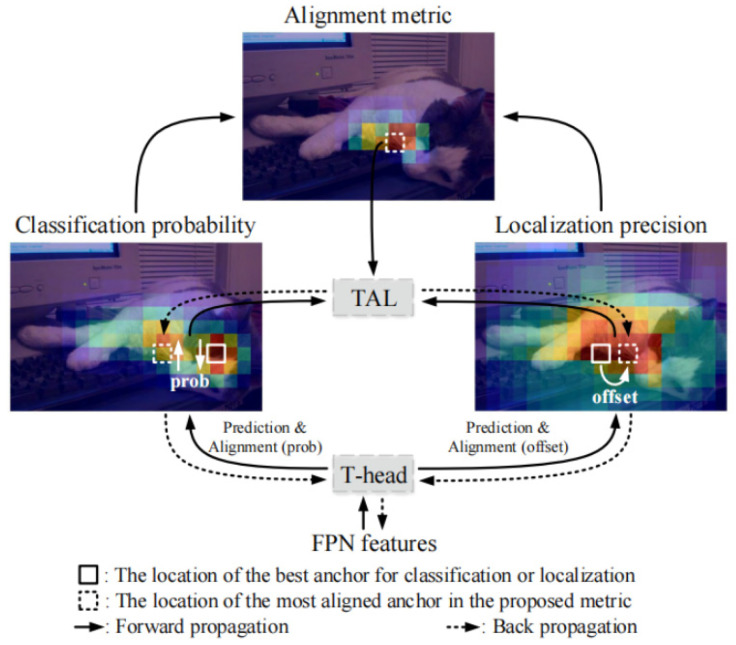
Overall learning mechanism of TOOD.

The original TOOD algorithm uses ResNet50 [26] for feature extraction and employs a Feature Pyramid Network (FPN) to integrate semantic information at different scales. Unlike traditional models with separate branches for classification and localization, TOOD features a unique task-pair flush (T-Head) structure (Figure 3). This structure enhances the interaction between classification and localization tasks, ensuring prediction consistency. The T-Head has two main parts: the task feature extraction module and the Task-Aligned Predictor (TAP). The T-Head first processes the semantic information from the FPN to compute task interaction features. These features are then fed into two parallel TAP structures, which measure the alignment between classification and localization tasks.

**Figure 4:**
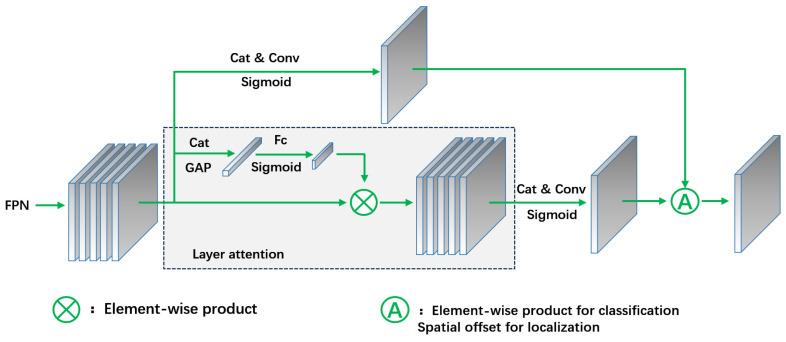
Diagram of TAP module structure.

The single branch design in task interaction features can lead to conflicts between classification and localization tasks due to their differing goals and focuses on hierarchy and perceptual fields [27]. In the TAP module, the layer attention mechanism processes task interaction features, dynamically optimizing them to improve classification and localization efficiency (Figure 4). The T-Head adjusts classification probability and localization prediction based on alignment metrics calculated by task alignment learning (TAL) during backpropagation, enabling it to provide expressive multi-scale features for both tasks.

### 2.4. Improvement of Feature Extraction Network

**Figure 5:**
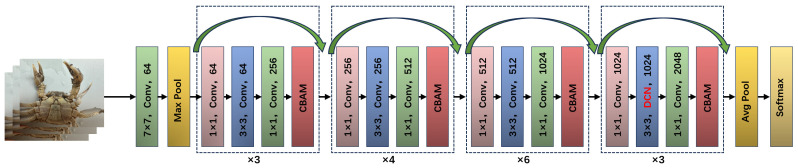
Illustration of the improved ResNeXt-50 network structure.

To enhance algorithm performance and address the issue of ResNet50 not effectively distinguishing between feature channels during feature fusion, this paper explores using an improved ResNeXt-50 as the feature extraction network [28]. Each residual unit of ResNeXt-50 incorporates a CBAM module [29], which adaptively recalibrates based on the importance of each feature channel, thereby enhancing the utilization of the residual units [30]. Additionally, given the variability in size and gender features of Chinese mitten crabs, a deformable convolutional network (DCN) replaces the standard convolution in the last residual block of ResNeXt-50 [31]. This adjustment allows the network to adapt to target variations through trainable offsets, significantly improving the robustness of target detection. The modified ResNeXt-50 network structure is depicted in Figure 5.

#### 2.4.1. ResNeXt Network

**Figure 6:**
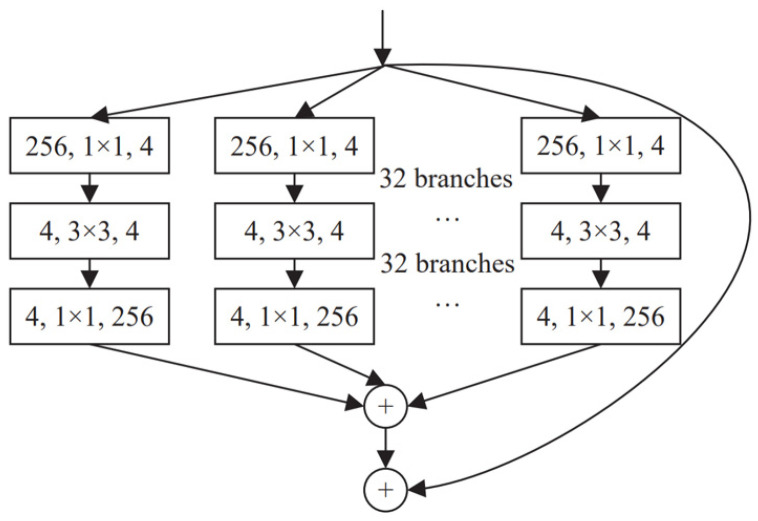
Basic module structure of ResNeXt.

ResNeXt, introduced in 2017, innovates CNN architecture with grouped convolution, dividing input into subsets for independent convolution operations before merging outputs. This approach broadens the network structure without increasing its depth or complexity. The basic ResNeXt module divides the convolution kernel into 32 groups, reducing input feature maps to 4 channels, processing with 3 × 3 convolution, and then increasing the channels to 256 and summing the results with residual connections (Figure 6). This method reduces model parameters while enhancing representation and generalization performance, making ResNeXt suitable for complex data tasks like identifying one-year-old precocious Chinese mitten crabs.

#### 2.4.2. Convolutional Block Attention Module

**Figure 7:**
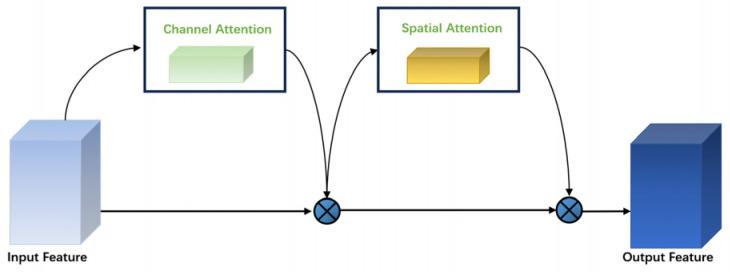
Illustration of the network structure of CBAM.

Attention mechanisms [32], which are crucial in deep learning for computer vision, are exemplified by the Convolutional Block Attention Module (CBAM). The CBAM enhances model performance by emphasizing key parts of the input feature map. It consists of two sub-modules: the Channel Attention Module (CAM) for identifying important channel features and the Spatial Attention Module (SAM) for highlighting relevant locations in the feature map. This sequential processing improves the model’s focus on task-relevant features (Figure 7).

The Channel Attention Module (CAM) of the CBAM uses global average and maximum pooling to extract channel-wide information and learn inter-channel dependencies, creating a channel attention map. The Spatial Attention Module (SAM) employs these pooling values to highlight important spatial locations, producing spatial attention maps through small convolutional operations. The CBAM’s main advantages are its versatility and minimal computational impact, allowing seamless integration into various CNN architectures, like ResNeXt. Embedding a CBAM in ResNeXt’s residual blocks enhances feature extraction [33,34], enabling deeper learning of male and female precocious crab features while reducing disturbances such as lighting, background, and angle, thus improving the model’s adaptability.

#### 2.4.3. Deformable Convolutional Networks

**Figure 8:**
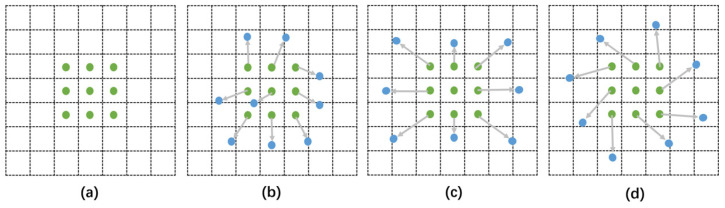
Illustration of the sampling locations in 3 × 3 standard and deformable convolutions. (**a**) The conventional sampling method of standard convolution. (**b**) Convolutional kernel with added offset. (**c**) Horizontal and vertical transformations of convolutional kernel after adding offsets. (**d**) Rotation transformation of convolution kernel after adding offset.

In traditional ResNeXt models, convolution samples are in fixed positions on the feature map, limiting its ability to handle geometric variations from occlusions, distance changes, etc., which affects localization accuracy. Deformable convolutional kernels, however, adjust dynamically based on the current image content, allowing sampling points to adaptively shift positions in response to the size and position changes of targets like Chinese mitten crabs against varying backgrounds. This method involves learning offsets for the sampling points, enabling the convolution kernel to focus on regions or objects of interest rather than fixed locations. Despite adding minimal parameter and computational overheads, deformable convolution significantly enhances target detection accuracy (Figure 8).

Ordinary 2D convolution consists of two key steps: first, the input feature map x is sampled over a regular grid R, and subsequently, the sampled values are weighted with the convolutional layer parameters w. The grid R defines the size and degree of expansion of the receiver domain, as shown in Equation (1):

(1) R={(−1,−1),(−1,0),…,(0,1),(1,1)}

Here, a 3 × 3 convolution kernel with a dilation of 1 is defined. In the standard convolution process, each position $y(p_{0})$ of the output feature map y is computed as shown in Equation (2):

(2) $$y(p_{0})=\sum_{p_{n}\in R}w(p_{n})\cdot x(p_{0}+p_{n})$$

where $p_{n}$ exhausts all the sampled positions in the regular grid R, while $p_{0}$ denotes each position in the input feature map for extracting semantic information near it.

And, in deformed convolution, the regular grid R is augmented with the offset $\left\{\Delta p_{n}|n=1,\ldots ,N\right\}$, where N=|R|, and the above formula has been changed to Equation (3):

(3) $$y(p_{0})=\sum_{p_{n}\in R}w(p_{n})\cdot x(p_{0}+p_{n}+\Delta p_{n})$$

In the formula, $\Delta p_{n}$ denotes the offset of the sampling point.

It can be seen that deformable convolution introduces an offset $\Delta p_{n}$ of the sampling point on top of the traditional convolution operation, thus adjusting the sampling positions of the key elements. Now, the sampling occurs at the irregular and offset positions $p_{n}+\Delta p_{n}$. Since the offset $\Delta p_{n}$ is usually of a fractional order, methods such as bilinear interpolation need to be used to obtain the corresponding values.

(4) $$y(p_{0})=\sum_{n=1}^{n}w(p_{n})\cdot x(p_{0}+p_{n}+\Delta p_{n})\cdot \Delta m_{n}$$

**Figure 9:**
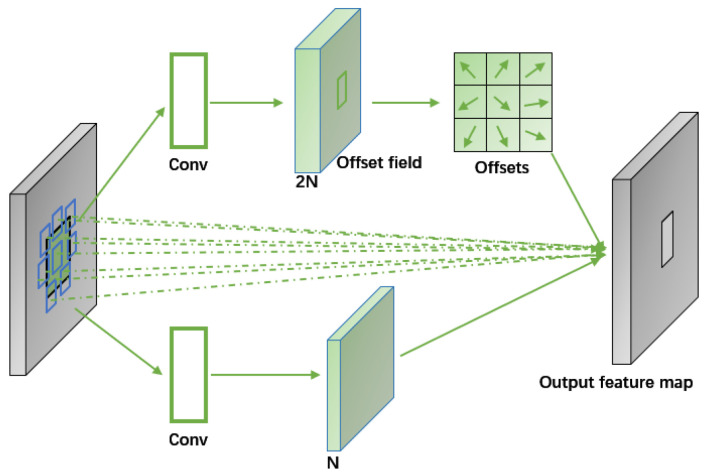
Realization of deformable convolution network.

In this equation, $\Delta m_{n}$ denotes the weight of each offset point, and its value range is [0, 1]. The input feature mapping contains N channels, and the number of channels corresponding to the offset portion of the sampling points is 2N (Figure 9). Because of the introduction of the weight coefficients, an additional N channels are added as the number of channels of the weighting network, and therefore, the number of channels of the final prediction result totals to 3N.

In this experiment, the feature pyramid structure utilizes a bottom-up fusion approach [35], closely linking feature extraction with the output of underlying residual blocks to improve adaptability to various sensory field sizes, thus enhancing the model’s localization accuracy. However, integrating numerous deformable convolutions increases the model’s complexity and may slow down the detection speed. To balance speed and accuracy, this study replaces the standard 3 × 3 convolution in the last residual block of ResNeXt with deformable convolution. This adjustment optimizes the balance between the parameter count and effective feature extraction.

### 2.5. Improvement of Feature Fusion Module

#### 2.5.1. ASFPN

**Figure 10:**
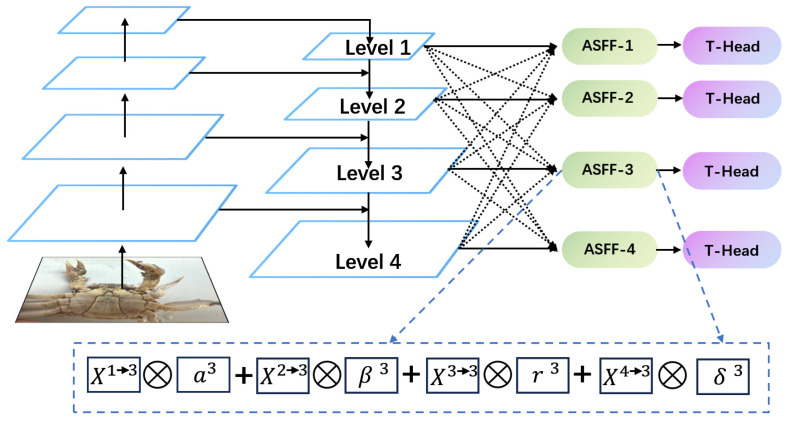
FPN structure diagram after integrating ASFF module.

Feature Pyramid Networks (FPNs) are commonly used for multi-scale fusion in deep convolutional neural networks to address multi-scale challenges in target detection [36]. Traditional FPNs up-sample high-level feature maps and fuse them with low-level maps, introducing high-level semantic information but often losing detail, particularly for small targets, due to the low resolution and limitations of the up-sampling process. Additionally, the typical methods of fusing feature maps through addition or splicing may not adequately preserve or utilize the detail information of small targets. To enhance the detection of small targets like the Chinese mitten crab in this experiment, we replaced the PA-Net in the feature fusion layer with an ASFF-based network [37]. This adjustment focused more clearly on key information and improved the detection performance of the samples (Figure 10).

#### 2.5.2. Principle of ASFF Module

Adaptive spatial feature fusion (ASFF) targets specific layers which vary in resolution and channel number within the feature fusion pyramid. ASFF first standardizes the resolution and channel number across different layers and then integrates these features to optimize the fusion strategy. This process adaptively fuses different layers, effectively filtering out conflicting information while retaining and enhancing discriminative details. For example, in ASFF-3, the fused feature map is produced by applying the learned weights a^3^, β^3^, r^3^, and δ^3^ to multiply and sum features from Level 1, Level 2, Level 3, and Level 4. The computational formula is outlined in Equation (5), and the general flow of ASFF is illustrated in the dashed box of Figure 10.

(5) $$y_{ij}^{l}=\alpha_{ij}^{l}\times x_{ij}^{1\to l}+\beta_{ij}^{l}\times x_{ij}^{2\to l}+\gamma_{ij}^{l}\times x_{ij}^{3\to l}+\delta_{ij}^{l}\times x_{ij}^{4\to l}$$

In this equation, $y_{ij}^{l}$ denotes the (i,j)-th vector of the output feature mapping $y^{l}$ between channels; $\alpha_{ij}^{l}$, $\beta_{ij}^{l}$, $\gamma_{ij}^{l}$, and $\delta_{ij}^{l}$ denote the learnable weights of the feature maps of the four different levels up to l. $x_{ij}^{1\to l}$, $x_{ij}^{2\to l}$, $x_{ij}^{3\to l}$, and $x_{ij}^{4\to l}$ denote the output of a certain output of the location feature map.

In computing the fused ASFF-3, we used a summation method, so that the Level 1 to Level 4 layers had to have the same feature size and number of channels in the summation. For this reason, we adjusted the number of channels by up-sampling or down-sampling the features at different levels. The feature maps of Level 1 to Level 4 were convolved at 1 s × 1 to obtain the weighting parameters a^3^, β^3^, r^3^, and δ^3^. These weighting parameters were spliced together and normalized by the Softmax function. The specific calculation formula is shown in Equation (6):

(6) $$\alpha_{ij}^{l}=\frac{e^{\lambda_{\alpha_{ij}}^{l}}}{e^{\lambda_{\alpha_{ij}}^{l}}+e^{\lambda_{\beta_{ij}}^{l}}+e^{\lambda_{\gamma_{ij}}^{l}}+e^{\lambda_{\delta_{ij}}^{l}}}$$

In this equation, $e^{\lambda_{\alpha_{ij}}^{l}}$, $e^{\lambda_{\beta_{ij}}^{l}}$, $e^{\lambda_{\gamma_{ij}}^{l}}$, and $e^{\lambda_{\delta_{ij}}^{l}}$ are the results obtained from the four feature maps after feature scaling using 1 × 1 convolution. These weight parameters satisfy $\alpha_{ij}^{l},\beta_{ij}^{l},\gamma_{ij}^{l},\delta_{ij}^{l}\in [0,1]$ and $\alpha_{ij}^{l}+\beta_{ij}^{l}+\gamma_{ij}^{l}+\delta_{ij}^{l}=1$.

### 2.6. Improvement of Head Detection

#### 2.6.1. T-Head Feature Alignment

The TOOD algorithm introduces the Task-Aligned Head (T-Head) to enhance one-stage object detection. Unlike Fully Convolutional One-Stage Object Detection (FCOS), which lacks a distinct localization branch and aligns tasks by weight sharing, the T-Head optimizes interaction between classification and localization. It calculates task interaction features, predicts alignment, and adjusts spatial predictions based on learning signals. The T-Head uses multiple convolutional layers to process FPN outputs, providing multi-level features and effective sensory fields for both tasks across scales. The specific formulas are detailed in Equation (7):

(7) $$X_{k}^{inter}=\left\{\begin{array}{c} \delta (conv_{k}(X^{fpn})),k=1\\ \delta (conv_{k}(X_{k-1}^{inter})),k>1 \end{array}\right.,\forall k\in \{1,2,\ldots ,N\}$$

The equation defines $conv_{k}$ as the k-th convolutional layer with ReLU activation δ. The TAL task alignment learning method establishes consistency metrics for classification and localization tasks, including anchor alignment and loss function, ensuring convergence. During backpropagation, the T-Head adjusts classification probabilities and localization predictions based on TAL’s learning signals, optimizing their distribution. A feature extractor learns task-interactive features from multiple convolutional layers, generating task interaction features.

#### 2.6.2. Anchor Alignment Metric

After T-Head and TAL processing, prediction results and feature alignment are explicitly output. To evaluate these predictions, an anchor alignment metric measures how well anchors align with the task, influencing sample allocation and refining anchor predictions dynamically. This metric prioritizes high-quality anchors based on classification scores and co-localization accuracy while reducing misaligned anchors. Equation (8) provides the mathematical expression for this metric:

(8) $$t=s^{p}+u^{q}$$

In this equation, s represents the classification score, u represents the IoU value, and p and q are weighting coefficients controlling the influence of classification and localization tasks on task alignment. The parameter t in Equation (8) jointly optimizes both tasks for classification and detection alignment. For each instance, the t of the generated anchors is calculated, keeping the maximum value as a positive sample and the other anchors as negative samples. TAL explicitly measures task alignment at the anchor level, filtering out anchors with the best performance in both tasks to achieve alignment.

The original algorithm enhances classification and localization interaction through the T-Head and explicitly aligns tasks via TAL, improving alignment issues. In this experiment, focusing on detecting one-year-old sexually precocious Chinese mitten crabs, we adjusted p and q to prioritize IoU optimization in localization. Ultimately, we set p to 0.4 and q to 6 in Equation (8).

### 2.7. Evaluation Indicators

In order to accurately assess the model’s localization recognition and accuracy for one-year-old sexually mature Chinese mitten crabs, we needed to evaluate the corresponding metrics. For this purpose, we calculated the precision (P), recall (R), F1-score, and mAP to measure the model’s recognition and classification performance on the experimental samples. The higher the value of these metrics, the better the detection effect of the model. The specific formulas for each evaluation metric are as follows:

(9) $$P=\frac{TP}{TP+FP}$$

(10) $$R=\frac{TP}{TP+FN}$$

(11) $$AP=\int_{0}^{1}P\left(R\right)dR$$

(12) $$mAP=\frac{1}{M}\sum_{k=1}^{M}AP(k)$$

(13) $$F1=\frac{2\times P\times R}{P+R}$$

In the formulas, TP stands for true positive samples, FP stands for false positive samples; FN stands for false negative samples; M stands for the total number of categories of the detection target; and AP(k) stands for the average precision value of the k-th category.

## 3. Results and Analysis

### 3.1. Model Training and Results

**Figure 11:**
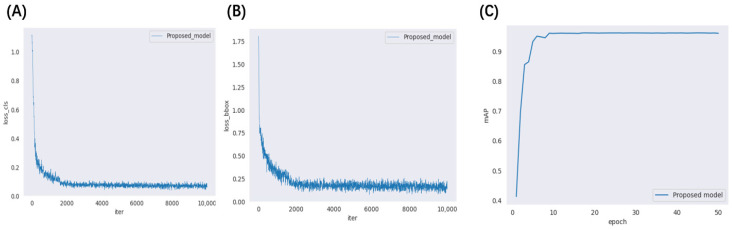
Illustration of the training results for R-TNET algorithm: (**A**) loss_cls curve; (**B**) loss bbox curve; (**C**) mAP50 curve.

The models are trained uniformly using self-constructed datasets and employ stochastic gradient descent (SGD) for gradient updating. Training involves 50 rounds, each with 100 batches. As shown in Figure 11, in (A) and (B), both loss_cls and loss_bbox decrease rapidly, particularly in the first 2000 iterations, eventually converging to 0.1. By 15 rounds, the loss functions stabilize, indicating well-trained models and rapid loss convergence. In (C), the mAP50 metric reaches 93.4% after about 10 rounds of training, ultimately achieving 96.1% on the test dataset. This demonstrates efficient training and high model performance.

### 3.2. Ablation Experiment

#### 3.2.1. Comparison of Different Feature Extraction Network Modules

We enhanced feature extraction for hard-to-recognize individuals by designing a more complex feature extraction network. Integrating these modules and comparing them with the benchmark network ResNet50, we observed significant improvements in detection performance. The mAP50 metric increased by 3.41 percentage points, while mAP and mAP75 improved by 2.68 and 4.12 percentage points, respectively (Table 2). These results validate the effectiveness of our improvement measures in optimizing the final detection outcome.

#### 3.2.2. Comparison of Different Feature Fusion Network Modules

**Figure 12:**
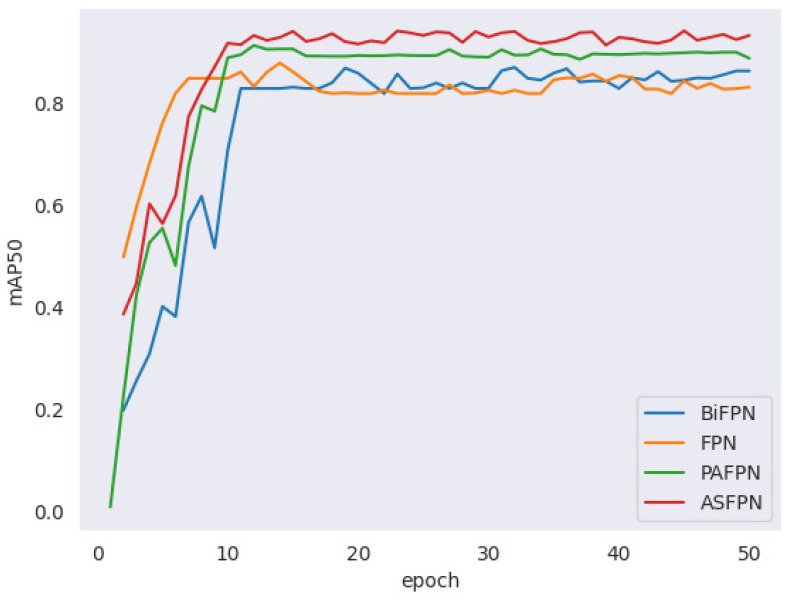
Plot of mAP50 values under different feature fusion methods.

The ASFPN module efficiently integrates and trains features, enhancing the accuracy of the localization and classification of one-year-old sexually precocious Chinese mitten crabs by fusing high-level semantic information and low-level features selectively and optimally. Comparative experiments with mainstream multi-scale feature fusion methods (e.g., FPN, PAFPN, and BiFPN) confirm the superiority of ASFPN. During the initial ten epochs, while all methods show a rapid increase in mAP50 metrics, ASFPN maintains stability and achieves excellent performance without a noticeable decrease in metrics (Figure 12).

### 3.3. Comparative Analysis with Other Models

In order to highlight the superiority of this paper’s algorithm in the target detection task of one-year-old precocious Chinese mitten crabs, we compared our R-TNET model with other mainstream target detection algorithms, including Faster RCNN, SSD, YOLOv5, and Cascade-RCNN, using the same experimental settings and datasets. R-TNET outperformed these algorithms on our self-built dataset, achieving an 88.78% mAP and a 96.14% mAP50. Specifically, it surpassed YOLOv5 and Cascade-RCNN by 4.19 and 5.43 percentage points in mAP50, respectively (Table 3). R-TNET’s F1-Score exceeded Faster RCNN, SSD, YOLOv5, and Cascade-RCNN by 3.1, 5.44, 1.77, and 2.65 percentage points, respectively. These results demonstrate R-TNET’s effectiveness in addressing challenges in one-year-old precocious Chinese mitten crab detection, significantly improving recognition accuracy to meet production needs.

In summary, our method outperforms mainstream target detection methods that use various backbone networks, achieving higher accuracy and significant advantages in both F1-scores and mAP indexes compared to the other four methods discussed.

### 3.4. Model Visualization Analysis

**Figure 13:**
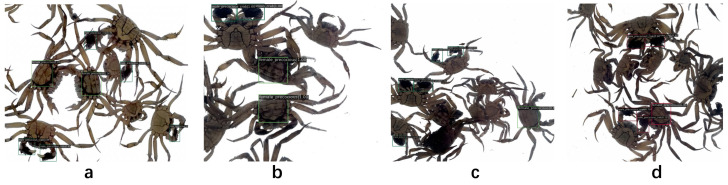
Detection results of one-year-old precocious Chinese mitten crabs (**a**–**d**).

To realistically simulate the detection effect of the model, we recorded a new video of the crab selection process, extracting 200 key frames for analysis using optimally trained weights. The results demonstrate that our algorithm effectively handles complex backgrounds and overlapping crab shells, maintaining robust performance even under challenging conditions (Figure 13). This is particularly true in image (d), where despite severe occlusion and lower confidence levels, the model accurately identified targets, meeting basic operational requirements. Additionally, it is worth noting that our model only takes 6 s to process these data, which is many times faster than manual processing. In terms of accuracy, the number of individuals who missed or missed detections was significantly lower than expected. Ultimately, this model was completed with high accuracy in a very short time, which required a long time for professionals to work on.

## 4. Discussion

In this paper, a task-aligned R-TNET target detection algorithm was proposed for the difficult problem of recognizing and selecting one-year-old precocious crabs in Chinese mitten crab cultures, aiming at solving the problems of difficult recognition and time-consuming and laborious selection. The experimental results showed that these improvements significantly enhance the detection accuracy of one-year-old precocious Chinese mitten crabs, and the mAP50 index reached 96.14%, which is far beyond the current mainstream target detection algorithms. Meanwhile, in order to meet the actual needs of farmers, we carried out on-site shooting and processing to verify the visualization and identification effect of the model on one-year-old sexually precocious crab juveniles in practical applications, and the results were in line with expectations.

## 5. Conclusions

The algorithm not only detects and classifies one-year-old precocious crab juveniles but also aids in quality grading and quantity prediction during selection. This method shows promise for automating Chinese mitten crab cultivation, reducing labor costs, and boosting economic returns in aquaculture. However, acquiring image data of crabs’ specific features remains challenging, necessitating specialized engineering equipment. Continuous optimization of our model is crucial to enhance its practicality and effectiveness in real farming scenarios.

## Figures and Tables

**Table 1:** Dataset details of crabs.

Dataset	Original Number	Number after Expansion	Training Set	Test Set
Image/piece	700	3928	3535	393
Male/piece	846	6465	5818	647
Female/piece	928	6322	5689	633

**Table 2:** Comparison of recognition effects using different feature extraction networks.

Backbone Network	mAP (%)	mAP50 (%)	mAP75 (%)
ResNet50	78.43	83.45	80.22
ResNeXt + CBAM	79.98	85.56	82.79
ResNeXt + CBAM + DCN	81.11	86.86	84.34

**Table 3:** Comparison of recognition effects of different detection models on self-built datasets.

Algorithm	Backbone	mAP (%)	mAP50 (%)	mAP75 (%)	F1-Score (%)
**Faster R-CNN**	ResNet-50 + BiFPN	84.88	89.29	91.45	94.79
**SSD**	SSDVGG + FPN	78.19	85.59	83.64	92.45
**YOLOv** **7**	ResNet-101 + PAFPN	84.61	91.19	87.97	96.12
**Cascade-RCNN**	ResNet-101 + FPN	82.36	90.71	85.67	95.24
**R-TNET**	ResNeXt + ASFPN	88.78	96.14	92.56	97.89

## Data Availability

The data presented in this study are available on request from the corresponding authors.

## References

[1] Zhang G., Jiang X., Zhou W., Chen W., Levy T., Wu X. (2024). Stocking density affects culture performance and economic profit of adult all-female Chinese mitten crabs (*Eriocheir sinensis*) reared in earthen ponds. Aquaculture.

[2] Pan Y., Liu C., Hong Y., Li Y., Yang H., Lin B., Dong Z., Lou Y., Fu S. (2024). Natural versus artificial seawater: Impacts on antioxidant capacity, muscle quality and gut microbiota of *Acanthopagrus schlegelii* during temporary rearing. Aquaculture.

[3] Zeng J., Tian J., Kan D., Han C., Fei X., Ma X., Wang Z., Shen W., Yang J., Ge J. (2023). Study of filtration, digestion and absorption of Chlorella pyrenoidosa by juvenile Chinese mitten crab (*Eriocheir sinensis*). Aquaculture.

[4] Chang G., Wu X., Cheng Y., Zeng C., Yu Z. (2017). Reproductive performance, offspring quality, proximate and fatty acid composition of normal and precocious Chinese mitten crab *Eriocheir sinensis*. Aquaculture.

[5] Wu X., He J., Jiang X., Liu Q., Gao F., Cheng Y. (2018). Does the wild-caught Chinese mitten crab megalopae perform better than the hatchery-produced seed during the juvenile culture?. Aquac. Res..

[6] Wang L., Gao J., Cao X., Du J., Cao L., Nie Z., Xu G., Dong Z. (2023). Integrated analysis of transcriptomics and metabolomics unveil the novel insight of one-year-old precocious mechanism in the Chinese mitten crab, *Eriocheir sinensis*. Int. J. Mol. Sci..

[7] Wu H., Ge M., Zhou X., Jiang S., Lin L., Lu J. (2019). Nutritional qualities of normal and precocious adult male Chinese mitten crabs (*Eriocheir sinensis*). Aquac. Res..

[8] Cheng H., Wu H., Liang F., Ge M., Jiang S., Lin L., Lu J. (2021). Comparison of the nutritional quality of three edible tissues from precocious and normal adult female Chinese mitten crabs (*Eriocheir sinensis*). J. Aquat. Food Prod. Technol..

[9] Fu C., Li F., Wang L., Wang A., Yu J., Wang H. (2019). Comparative transcriptology reveals effects of circadian rhythm in the nervous system on precocious puberty of the female Chinese mitten crab. Comp. Biochem. Physiol. Part D Gen. Proteomics.

[10] Liu H., Guo L., Zhang W., Peng J., Chen Q., Cao F., Zhang Z., Guo M., Zhang H., Mu S. (2022). Transcriptome and proteome reveal abnormal spermatozoa in precocious Chinese mitten crab, *Eriocheir sinensis*. Aquac. Rep..

[11] Zhang Z., Xiao J., Wang W., Zielinska M., Wang S., Liu Z., Zheng Z. (2024). Automated grading of angelica sinensis using computer vision and machine learning techniques. Agriculture.

[12] Chaurasia D., Patro B.D.K. (2024). Detection of objects in satellite and aerial imagery using channel and spatially attentive YOLO-CSL for surveillance. Image Vis. Comput..

[13] Zhao D., Liu X., Sun Y., Wu R., Hong J., Ruan C. (2019). Detection of underwater crabs based on machine vision. Trans. Chin. Soci. Agric. Mach..

[14] Zhao D., Cao S., Sun Y., Qi H., Ruan C. (2020). Small-sized efficient detector for underwater freely live crabs based on compound scaling neural network. Trans. Chin. Soci. Agric. Mach..

[15] Cui Y., Pan T., Chen S., Zou X. (2020). A gender classification method for Chinese mitten crab using deep convolutional neural network. Mult. Tools Appl..

[16] Wei T., Zheng X., Li T., Wang R. (2024). Multi-group convolutional neural network for gender recognition of *Portunus tritubereulatus*. South China Fish. Sci..

[17] Cao Y., Ren K., Chen Q. (2023). Template matching based on convolution neural network for UAV visual localization. Optik.

[18] Chen X., Zhang Y., Li D., Duan Q. (2023). Chinese mitten crab detection and gender classification method based on GMNet-YOLOv4. Comput. Electron. Agric..

[19] Gu H., Chen M., Gan D. (2024). Gender identification of Chinese mitten crab juveniles based on improved Faster R-CNN. Appl. Sci..

[20] Ahmed A., Imran A.S., Manaf A., Kastrati Z., Daudpota S.M. (2024). Enhancing wrist abnormality detection with YOLO: Analysis of state-of-the-art single-stage detection models. Biomed. Signal Process. Control.

[21] Liu H., Wang X., Zhao F., Yu F., Lin P., Gan Y., Ren X., Chen Y., Tu J. (2024). Upgrading swin-B transformer-based model for accurately identifying ripe strawberries by coupling task-aligned one-stage object detection mechanism. Comput. Electron. Agric..

[22] Safdar M., Li Y.F., El Haddad R., Zimmermann M., Wood G., Lamouche G., Wanjara P., Zhao Y.F. (2024). Accelerated semantic segmentation of additively manufactured metal matrix composites: Generating datasets, evaluating convolutional and transformer models, and developing the MicroSegQ+ Tool. Expert. Syst. Appl..

[23] Torralba A., Russell B.C., Yuen J. (2010). LabelMe: Online image annotation and applications. Proc. IEEE.

[24] Yang N., Zhao Y., Chen J., Wang F. (2023). Real-time classification for Φ-OTDR vibration events in the case of small sample size datasets. Opt. Fiber Technol..

[25] Pu H., Xu K., Zhang D., Liu L., Liu L., Wang D. (2022). TA-BiDet: Task-aligned binary object detector. Neurocomputing.

[26] Wang H., Ying J., Liu J., Yu T., Huang D. (2024). Harnessing ResNet50 and SENet for enhanced ankle fracture identification. BMC Musculoskel. Disord..

[27] Guo X., Guo X., Zou Q., Wulamu A., Yang M., Zheng H., Guo X., Zhang T. (2023). FE-trans-net: Feature enhancement based single branch deep learning model for surface roughness detection. J. Manuf. Processes.

[28] Gaur A., Pant G., Jalal A.S. (2023). Comparative assessment of artificial intelligence (AI)-based algorithms for detection of harmful bloom-forming algae: An eco-environmental approach toward sustainability. Appl. Water Sci..

[29] Sangeetha S., Indumathi N., Grover R., Singh R., Mavi R. (2024). IoT based wireless communication system for smart irrigation and rice leaf disease prediction using ResNeXt-50. Inter. J. Artif. Intell. Tools.

[30] Tie J., Wu W., Zheng L., Wu L., Chen T. (2024). Improving walnut images segmentation using modified UNet3+ algorithm. Agriculture.

[31] Shin Y., Shin H., Ok J., Back M., Youn J., Kim S. (2024). DCEF2-YOLO: Aerial detection YOLO with deformable convolution–efficient feature fusion for small target detection. Remote Sens..

[32] Li H., Ma Z., Xiong S.-H., Sun Q., Chen Z.-S. (2024). Image-based fire detection using an attention mechanism and pruned dense network transfer learning. Inf. Sci..

[33] Xu Y., Li J., Zhang L., Liu H., Zhang F. (2024). CNTCB-YOLOv7: An effective forest fire detection model based on ConvNeXtV2 and CBAM. Fire.

[34] Zeng W., He M. (2024). Rice disease segmentation method based on CBAM-CARAFE-DeepLabv3+. Crop Prot..

[35] Michopoulos J.G., Iliopoulos A.P., Farhat C., Avery P., Daeninck G., Steuben J.C., Apetre N.A. (2024). Bottom-up hierarchical and categorical metacomputing for automating composition and deployment of directly computable multiphysics models. J. Comput. Sci..

[36] Luo Q., Shao J., Dang W., Wang C., Cao L., Zhang T. (2024). An efficient feature pyramid attention network for person re-identification. Image Vis. Comput..

[37] Shaheema S.B., Muppalaneni N.B. (2024). Explainability based panoptic brain tumor segmentation using a hybrid PA-NET with GCNN-ResNet50. Biomed. Signal Process. Control.

